# Supplementary material for: HIVprotI: an integrated web based platform for prediction and design of HIV proteins inhibitors
Source: J Cheminform. 2018 Mar 9;10:12. doi: 10.1186/s13321-018-0266-y (PMC5845081; doi:10.1186/s13321-018-0266-y)
Supplement: Supplementary file 2 — Additional file 2. Source code of HIVProtI web server. [file 13321_2018_266_MOESM2_ESM.zip › HIVprotI_Source-code/hivprotI/weka-3-6-6/doc/weka/gui/beans/ThresholdDataEvent.html]

ThresholdDataEvent


---


|  |  |  |
| --- | --- | --- |
| **Overview** \| **Package** \| **Class** \| **Tree** \| **Deprecated** \| **Index** \| **Help** \| **Weka's home** | |  |
| **PREV CLASS**   **NEXT CLASS** | **FRAMES**    **NO FRAMES**     **All Classes** |  |
| SUMMARY: NESTED \| FIELD \| CONSTR \| METHOD | DETAIL: FIELD \| CONSTR \| METHOD |  |


---


## weka.gui.beans Class ThresholdDataEvent

```
java.lang.Object
  java.util.EventObject
      weka.gui.beans.ThresholdDataEvent
```

**All Implemented Interfaces:**: java.io.Serializable

---

``` public class ThresholdDataEvent extends java.util.EventObject ```

Event encapsulating classifier performance data based on
varying a threshold over the classifier's predicted probabilities

**Version:**
:   $Revision: 5751 $

**Author:**
:   Mark Hall

**See Also:**: `EventObject`, Serialized Form

---

**Constructor Summary**

`ThresholdDataEvent(java.lang.Object source,
PlotData2D dataSet)`

`ThresholdDataEvent(java.lang.Object source,
PlotData2D dataSet,
Attribute classAtt)`


| **Method Summary** | |
| --- | --- |
| `Attribute` | `getClassAttribute()`             Return the class attribute for which the threshold data was generated for. |
| `PlotData2D` | `getDataSet()`             Return the instances of the data set |

**Methods inherited from class java.util.EventObject**

`getSource, toString`

**Methods inherited from class java.lang.Object**

`equals, getClass, hashCode, notify, notifyAll, wait, wait, wait`

**Constructor Detail**

### ThresholdDataEvent

```
public ThresholdDataEvent(java.lang.Object source,
                          PlotData2D dataSet)
```

---


### ThresholdDataEvent

```
public ThresholdDataEvent(java.lang.Object source,
                          PlotData2D dataSet,
                          Attribute classAtt)
```


**Method Detail**

### getDataSet

```
public PlotData2D getDataSet()
```

:   Return the instances of the data set

    :   **Returns:**: an `Instances` value

---


### getClassAttribute

```
public Attribute getClassAttribute()
```

:   Return the class attribute for which the threshold data was generated
    for.

    :   **Returns:**: the class attribute for the threshold data or null if not set.


---


|  |  |  |
| --- | --- | --- |
| **Overview** \| **Package** \| **Class** \| **Tree** \| **Deprecated** \| **Index** \| **Help** \| **Weka's home** | |  |
| **PREV CLASS**   **NEXT CLASS** | **FRAMES**    **NO FRAMES**     **All Classes** |  |
| SUMMARY: NESTED \| FIELD \| CONSTR \| METHOD | DETAIL: FIELD \| CONSTR \| METHOD |  |


---
